# Exome sequencing in undiagnosed congenital myopathy reveals new genes and refines genes–phenotypes correlations

**DOI:** 10.1186/s13073-024-01353-0

**Published:** 2024-07-09

**Authors:** Yvan de Feraudy, Marie Vandroux, Norma Beatriz Romero, Raphaël Schneider, Safaa Saker, Anne Boland, Jean-François Deleuze, Valérie Biancalana, Johann Böhm, Jocelyn Laporte

**Affiliations:** 1 IGBMC, Inserm U1258, Cnrs UMR7104, Université de Strasbourg, 1 Rue Laurent Fries, Illkirch, 67404 France; 2 Department of Pediatric Neurology, CHU Strasbourg, Strasbourg, France; 3 Centre de Référence Neuromusculaire Nord-Est-Île de France, Strasbourg, France; 4 Myology Institute, Neuromuscular Morphology Unit, Sorbonne Université, INSERM, GHU Pitié-Salpêtrière, Paris, France; 5 Genethon, DNA and Cell Bank, Evry, 91000 France; 6 Centre National de Recherche en Génomique Humaine (CNRGH), Université Paris-Saclay, CEA, Evry, 91057 France; 7 Laboratoire de Diagnostic Génétique CHRU de Strasbourg, Strasbourg, 67091 France

**Keywords:** Congenital myopathy, Myopathy, Exome sequencing, Genetic diagnosis, Genetic heterogeneity, Phenotypic heterogeneity, Core myopathy, Nemaline myopathy, Centronuclear myopathy, Tubular aggregate myopathy

## Abstract

**Background:**

Congenital myopathies are severe genetic diseases with a strong impact on patient autonomy and often on survival. A large number of patients do not have a genetic diagnosis, precluding genetic counseling and appropriate clinical management. Our objective was to find novel pathogenic variants and genes associated with congenital myopathies and to decrease diagnostic odysseys and dead-end.

**Methods:**

To identify pathogenic variants and genes implicated in congenital myopathies, we established and conducted the MYOCAPTURE project from 2009 to 2018 to perform exome sequencing in a large cohort of 310 families partially excluded for the main known genes.

**Results:**

Pathogenic variants were identified in 156 families (50%), among which 123 families (40%) had a conclusive diagnosis. Only 44 (36%) of the resolved cases were linked to a known myopathy gene with the corresponding phenotype, while 55 (44%) were linked to pathogenic variants in a known myopathy gene with atypical signs, highlighting that most genetic diagnosis could not be anticipated based on clinical–histological assessments in this cohort. An important phenotypic and genetic heterogeneity was observed for the different genes and for the different congenital myopathy subtypes, respectively. In addition, we identified 14 new myopathy genes not previously associated with muscle diseases (20% of all diagnosed cases) that we previously reported in the literature, revealing novel pathomechanisms and potential therapeutic targets.

**Conclusions:**

Overall, this approach illustrates the importance of massive parallel gene sequencing as a comprehensive tool for establishing a molecular diagnosis for families with congenital myopathies. It also emphasizes the contribution of clinical data, histological findings on muscle biopsies, and the availability of DNA samples from additional family members to the diagnostic success rate. This study facilitated and accelerated the genetic diagnosis of congenital myopathies, improved health care for several patients, and opened novel perspectives for either repurposing of existing molecules or the development of novel treatments.

**Supplementary Information:**

The online version contains supplementary material available at 10.1186/s13073-024-01353-0.

## Background

Congenital myopathies (CM) are rare and severe genetic diseases strongly impacting patient autonomy and often survival. To date, many patients do not have a genetic diagnosis, precluding a better health care, the application of potential treatments, and genetic counseling including prenatal diagnosis [1–3]. Here we aimed to decrease diagnostic odysseys or dead-ends and to identify novel causative genes which will represent novel therapeutic targets.

CM are linked to muscle weakness and/or hypotonia, usually at birth or starting in early infancy, albeit some adult-onset cases were described [4–6]. Additional signs as cardiomyopathy, orthopedic deformities, or respiratory failure are also often noted. CM are not associated with muscle necrosis and regeneration and thus differ from muscular dystrophies. Instead, muscle biopsies from CM patients typically show structural anomalies characteristic for the different CM subtypes. The main subtypes are core myopathy (central core disease and multi-minicore disease), nemaline myopathy (NM) with protein aggregates forming rod-like structure, and centronuclear and myotubular myopathies with mis-localized nuclei and organelles. Less frequently, tubular aggregate, cylindrical spirals, hexagonally cross-linked tubular arrays, or other structural anomalies can be observed. Over the past 10 years, the number of causative CM genes increased from 19 to 47 genes and most code for myofilaments or proteins regulating calcium-coupled muscle contraction [7, 8].

Before 2012, the genetic diagnosis was performed on a gene-by-gene basis suggested by clinical and histopathological findings and limited by the knowledge of the implicated genes and sometimes hampered by the extreme size of some genes (*TTN, RYR1, NEB*). In 2012, gene panels targeting parts or all of the known neuromuscular genes were validated [9, 10]. Now, next-generation sequencing (NGS) as exome and genome sequencing is usually conducted in routine diagnosis laboratories for patients excluded for frequent genetic alterations through targeted PCR (i.e., *DMPK*) or enzymatic activity.

Here, to identify pathogenic variants and genes implicated in CM, we assembled a large cohort of 310 families classified into homogeneous CM subtypes based on clinical and histopathological findings. DNA samples from probands and relatives were analyzed by exome sequencing and in-house bioinformatics tools and candidate variants/genes were confirmed through genetic and functional investigations. This study is part of the French MYOCAPTURE NGS project aiming to identify novel genes mutated in myopathies by sequencing the exome of > 1000 individuals from families with different forms of myopathies.

## Methods

### Patient recruitment and evaluation

The present project focused on congenital myopathies. Patients were examined by clinicians from the French neuromuscular network FILNEMUS (www.filnemus.fr) or from their country of origin. All patients enrolling the study manifested either neonatal hypotonia or early- or late-onset muscle weakness, and most underwent muscle biopsies with histological analysis. Most biopsies were ascertained through the Morphology Unit of the Myology Institute (Paris, France), which receives requests from all over France, as well as from South America, while the remaining biopsies were analyzed by histologists from the patient’s home countries. Patients were classified into homogeneous CM cohorts based on the clinical severity and histological findings on the muscle biopsies (Table 1). To enhance cohort homogeneity, the majority of histological classification was conducted by a single histopathologist (NBR, Myology Institute, Paris). Clinical, histological, and genetic data of patients are included in Table S1, where associated publications are mentioned when previously published.

**Table 1:** Patients cohorts based on clinical and histological findings. Main histological features on muscle biopsies and diagnostic success rate per group

Patient cohorts	Total number of cases: no. (%)	Number of cases with identified variants: no. (%)
Centronuclear myopathy	82 (26)	43 (52)
Core myopathy	54 (17)	21 (39)
Nemaline myopathy	41 (13)	20 (49)
Tubular aggregate myopathy	34 (10)	8 (24)
Myopathy with protein aggregates	13 (4)	3 (23)
Core-rod myopathy	8 (2)	6 (75)
Vacuolar myopathy	7 (2)	5 (71)
Cylindrical spirals	4 (1)	0 (0)
Protein aggregates and rimmed vacuoles	3 (1)	2 (67)
Cap myopathy	3 (1)	1 (33)
Cytoplasmic bodies	3 (1)	1 (33)
Abnormal nuclear envelop	3 (1)	0 (0)
Mini-rods myopathy	2 (1)	1 (50)
Congenital fiber type disproportion	1 (1)	1 (100)
Broad A band disease	1 (1)	1 (100)
Myofibrillar myopathy	1 (1)	0 (0)
Hexagonally cross-linked crystalloid inclusions	1 (1)	0 (0)
Dark inclusion	1 (1)	0 (0)
Undefined CM	19 (6)	6 (32)
No biopsy	29 (9)	4 (14)
Total	310 (100)	123 (40)

For most patients, the implication of the main known genes at the time of inclusion (2009–2018) was excluded mainly through targeted gene sequencing or rarely through myopathy-related gene panel. For *RYR1*, the 5’ sequence was barely tested, while RT-PCR was occasionally used. Based on these criteria, we collected DNA samples from 310 families, including 429 patients and 459 unaffected relatives, representing a varied ethnic population.

### Exome sequencing and data analysis

Library and exome capture of patients were performed with the SureSelect Human all exon kit (Agilent v2 or v5, Santa Clara, CA) and samples were paired-end sequenced on a HiSeq2000 (Illumina, San Diego, CA). Sequences were obtained from the CNRGH (Evry, France), the Genomeast platform (Illkirch-Graffenstaden, France), or BGI (Shenzhen, China). Sequence data were aligned to the reference genome GRCh37/hg19. Analysis of NGS data and variants calling was performed as previously described [11]. We applied segregation scenarios based on the family structure (recessive homozygous/compound heterozygous, dominant, de novo, X-linked, and other specific scenarios). Filtering of variants was also based on population frequency in both the gnomAD database and an internal NGS database. Then, we used our VaRank algorithm to weight and prioritize variant [12]. For missense variants, we used in silico predictions of pathogenicity with SIFT [13] or PolyPhen[14]. For splice variants, we used splice variant predictor tools with MaxEntScan [15], NNSplice [16], and Splice Site Finder [17]. For a subset of synonymous, canonical or non-canonical splice site variants, RT-PCR from muscle RNA with subsequent cDNA sequencing was used to attest a pathogenic effect on exon splicing. For certain variants, a segregation study was required to determine their pathogenicity. In the absence of DNA samples from relatives, these variants were classified as candidates. Variants were classified according to the ACMG standards and guidelines [18].

### Other investigations

In case of homozygous variants, familial consanguinity was assessed using Somalier, measuring the relatedness between two DNA samples by comparing informative genetic variations [19]. When a pathogenic variant was confirmed in a known gene, the clinical and histological phenotype of the patient was compared to those already described in the OMIM database to determine if it corresponded to a novel phenotype. For novel genes, functional validation of the pathogenicity of the variants was performed using in vitro experiments to attest their impact on RNA stability, protein level, and protein functions, while in vivo models were used to demonstrate they could reproduce patient phenotypes. Protocols used and results obtained have been detailed and published in the associated papers [20–29].

### Ethics

The present study was approved by the IRB Comité de Protection des Personnes Est IV (DC-2012–1693), and all participants or their legal guardians provided written informed consents to the different clinical centers involved in the project.

## Results

### Description of the cohort

**Fig. 1:**
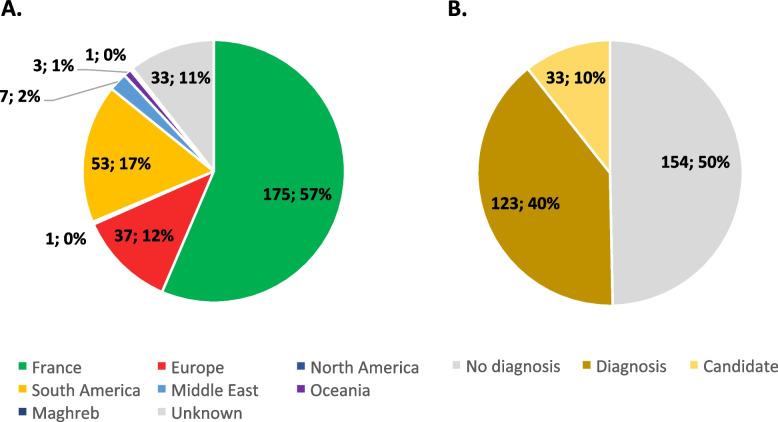
Overview of patients enrolling the MYOCAPTURE cohort. **A** Geographic origin of genetic samples. Europe: European countries other than France. **B** Diagnosis ratio. Diagnosis: patients with confirmed pathogenic variant(s). Candidate: patients with suspected pathogenic variant(s) in one candidate gene

A total of 310 families with genetically undiagnosed CM enrolled this study from 2009 to 2018. For 214 families, at least one relative was available, and for 92 families, a complete trio composed of the index patient and both parents was available. Overall, 429 individuals were analyzed by exome, or Sanger sequencing for segregation study. Most probands were sporadic cases (78%) with no other affected family member or known family history of a neuromuscular disorder, while familial cases with apparent X-linked, autosomal dominant, or autosomal recessive disease inheritance represented 22%. Parents were reported to be consanguineous in 25 families. A majority of 61% of the probands was male, while 39% were female. In this international multicentric study, 175 probands were from France (57%), 102 probands (32%) from 15 countries encompassing Algeria, Argentina, Australia, Belgium, Brazil, Canada, Finland, Germany, Israel, Italy, Luxembourg, New Zealand, Spain, Turkey, Uruguay, and 32 probands from unknown origin (11%) (Fig. 1A).

Age of onset was known for 99 probands (32%), with 58 probands manifesting antenatal or neonatal disease signs, 27 with childhood/adolescence onset, and 14 with onset in adulthood. This last category had the highest proportion of families with more than one affected individual. Muscle biopsies were performed and morphologically examined for 281 probands (91%). Based on the types of histological anomalies, families were classified into homogeneous cohorts (Table 1). Centronuclear myopathy (CNM) was the largest cohort (*n* = 82 cases, 26%), followed by core myopathy (*n* = 54, 17%), NM (*n* = 41, 13%), and tubular aggregate myopathy (TAM) (*n* = 34, 10%). This distribution most probably reflects an inclusion bias as our laboratory has a known expertise in CNM and TAM.

### Overall genetic characterization of the cohort

Exome sequencing identified causal pathogenic variants for 123 families (40%) (Fig. 1B). A comparative overview of the clinical, histological, and genetic findings of all families with confirmed molecular diagnosis is given in Table S1. For 33 families (10%), we detected sequence variants in convincing candidate genes, but DNA samples from relatives were not available, precluding segregation analyses. In our cohort, there was a correlation between the number of sequenced family members and the diagnosis rate. Increasing the number of sequenced family members enhanced the identification of the pathogenic variants, with a majority of families (51%) being diagnosed when at least three DNA samples were available (Fig S1). More specifically, the proportion of diagnosed families was higher for trios (48%) compared to duos (29%). Similarly, the proportion of families with identified pathogenic variants increased when the number of affected individuals per families increased, with a majority of families (55%) being diagnosed when at least two affected individuals were available (Fig S2). Muscle biopsies with histological examination were another major factor contributing to molecular diagnosis. Indeed, causative variants were identified in 42% of the families with biopsy and only in 14% of the families without biopsy (Fig S3). This may be partially due to the fact that patients with the most severe clinical phenotypes are more often biopsied; however, these results support the importance of complementary investigations in addition to clinical assessment, as histopathology (or MRI, not implemented in the MYOCAPTURE project), to precisely diagnose CM and prioritize candidate genes.

**Fig. 2:**
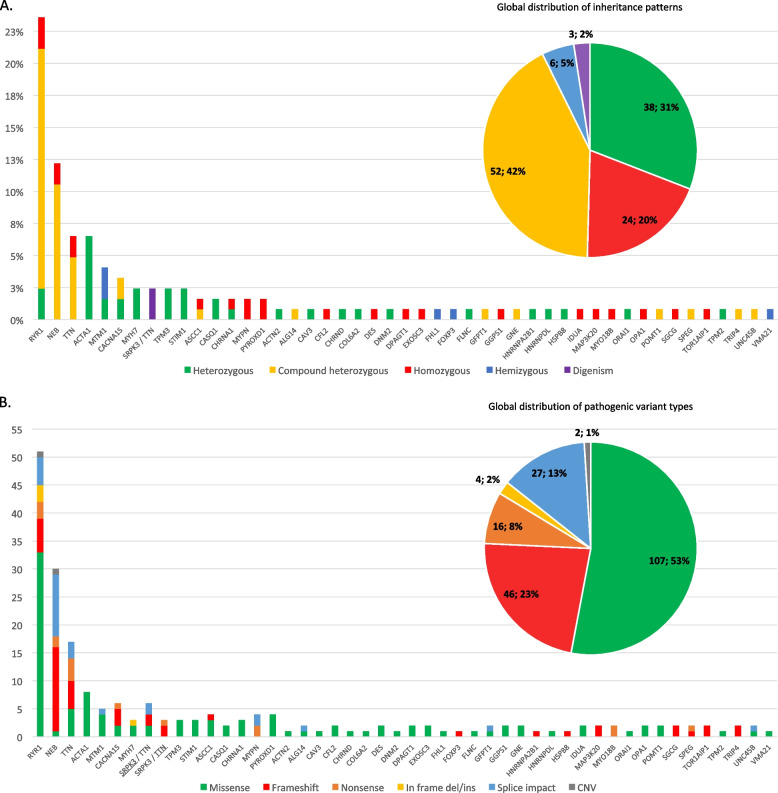
Distribution of inheritance patterns and types of pathogenic variants linked to the different implicated genes in the MYOCAPTURE cohort. **A** Mode of inheritance of pathogenic variants in diagnosed patients. The bar chart indicates the ratio of patients with pathogenic variants in a given gene. The color code illustrates the mode of inheritance per gene (bars) and as a global overview (diagram). **B** Type of pathogenic variants. The bar chart shows the number of pathogenic variants per gene. Homozygous mutations count as two. For the patients with *SRPK3*/*TTN* digenism, the pathogenic variants in *TTN* and *SRPK3* are depicted in separate bars. The respective gene is underlined. The overall distribution of pathogenic variant types is illustrated in the upper diagram. CNV copy number variants

The majority of families enrolling the MYOCAPTURE project displayed autosomal recessive pathogenic variants (62%), with 31% of the families exhibiting autosomal dominant pathogenic variants and 5% having X-linked pathogenic variants (Fig. 2A). The families with autosomal recessive disease inheritance either carried homozygous or compound heterozygous pathogenic variants. Only 14 patients with heterozygous, hemizygous, or compound heterozygous pathogenic variants carried de novo pathogenic variants (15%). This knowledge should be useful for genetic information of relatives. Patients from the 16 diagnosed consanguineous families did not always exhibit homozygous pathogenic variants, as one proband had a dominant pathogenic variant in the *STIM1* gene (family 105). This example confirms that the occurrence of de novo mutations is equally probable in consanguineous and non-consanguineous families and that consanguineous families should not only be screened for homozygous variants. Conversely, consanguinity was revealed in one family with homozygous pathogenic variant through the use of the Somalier software (family 33). In total and across all patient cohorts, we identified 202 pathogenic variants (Table S1) with a majority of missense variants (53%) and frameshift variants (23%) (Fig. 2B). The majority of the pathogenic variants are unique. Only 12 (6%) are shared by a maximum of 2 patients each, with 9 of them involving the *RYR1* gene. Thus, in this cohort, there was no strong mutation hotspot or founder effect. Finally, two probands (family 8 and 49) displaying different histological presentations (respectively NM and cap myopathy) were, in fact, third-degree relatives. They had pathogenic variants in different genes (respectively in *ACTA1* and *MYPN*). This highlights the possibility of the occurrence of two distinct and unrelated myopathies within large families.

### Main congenital myopathy genes and impact of exome sequencing

**Fig. 3:**
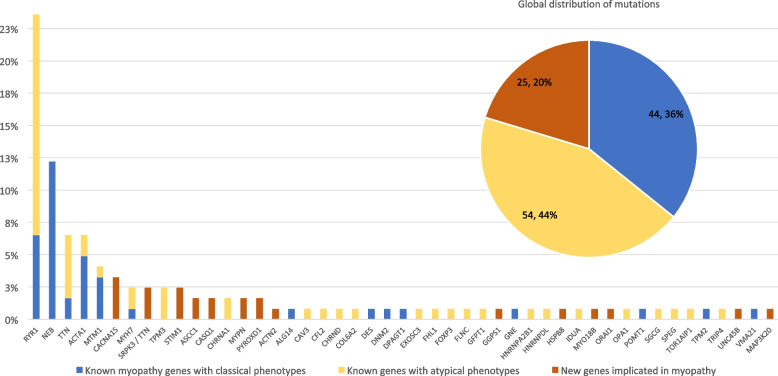
Distribution of known and novel myopathy genes. In the bar chart, the percentages indicate the ratio of families carrying pathogenic variant(s) in a given gene. New genes identified within the MYOCAPTURE project are shown in orange. For known genes, percentages of patients with classical phenotype (blue) or with new phenotype (yellow) are indicated. The global distribution of pathogenic variants is depicted in the upper diagram

Among the 123 families with confirmed molecular diagnosis, 47 different causative genes were identified. Strikingly, 4 genes were over-represented with diseased-associated variants in 60 of these families (49%). Specifically, the *RYR1* gene was implicated in the highest number of cases (*n* = 29 cases, 24%), followed by *NEB* (*n* = 15, 12%), *TTN* (*n* = 8, 7%), and *ACTA1* (*n* = 8, 7%) (Fig. 3).

Sixteen of the 123 diagnosed families (13%) carried pathogenic variants that may easily escape routine diagnosis. Seven pathogenic variants were identified in non-canonical splice sites in *MTM1* (*n* = 1), *RYR1* (*n* = 1), *UNC45B* (*n* = 1), and *NEB* (*n* = 4). Five synonymous *NEB*, *RYR1*, and *TTN* variants were found to impact on splicing in three families with NM, one family with core myopathy and one family with CNM, respectively. Three families with NM had mosaic pathogenic variants in the *ACTA1* gene that was detectable on muscle DNA and only barely detectable on lymphocyte DNA [30]. Finally, heterozygous pathogenic *MTM1* variants were detected in two symptomatic female carriers with CNM, and one *RYR1* missense variant previously considered as polymorphism has now been reclassified as a pathogenic variant in a CNM family [31]. Overall, these examples emphasize the importance to thoroughly examine non-coding regions, use prediction programs to evaluate potential pathogenic effects of intronic and synonymous variants, perform high-throughput sequencing on DNA from the affected tissue or adjust the bioinformatics pipeline filters to detect variants in a low number of reads, and consider X-linked disorders also in females.

Among the 123 diagnosed families, 11 (9%) had pathogenic variants that were not detected by previous myopathy-related gene panels or targeted sequencing of known CM genes. This was essentially due to the presence of pathogenic variants in the large *RYR1* gene (*n* = 8), which was previously only sequenced for the most 3’-terminal exons, or to mosaic pathogenic variants in *ACTA1* (*n* = 3), which were not detected by routine genetic tests on lymphocyte DNA. As a result, the use of exome sequencing on muscle DNA became necessary for one of these patients to identify the causative gene.

### Known myopathy genes linked to classical phenotypes

For 36% of the families (*n* = 44) with confirmed molecular diagnosis, we identified pathogenic variants in known CM genes concurring with the clinical and histopathological characteristics. The majority of these cases encompassed pathogenic *RYR1* variants in core myopathy, *NEB* and *ACTA1* in nemaline myopathy, and pathogenic *MTM1* variants in CNM (Fig. 3). As examples, eight patients with core myopathy had pathogenic variants in *RYR1*, eight NM cases harbored the typical compound heterozygous combination of a nonsense or a frameshift variant with a splice site variant affecting an in-frame exon of *NEB*. We also detected heterozygous missense variants in the highly conserved *ACTA1* gene in five patients with classical NM and nemaline rods on muscle sections. Also, one CNM case with severe neonatal hypotonia and the presence of abnormally centralized nuclei in the myofiber carried heterozygous missense variant affecting the PH domain of DNM2 [32].

### Genes linked to atypical phenotypes

In addition to pathogenic variants in known CM genes associated with a classical phenotype, we also found that the majority (51%) of pathogenic variants were located in previously known neuromuscular genes, including 44% in patients displaying atypical phenotypes at the time of inclusion (Fig. 3). They were divided into three categories:

- Pathogenic variants in known genes associated with a different muscle disorder: importantly, 46 of the 123 diagnosed families (37%) had pathogenic variants in genes previously associated with other forms of muscular diseases, with 40 families showing pathogenic variants in genes associated with other forms of myopathy (Table 2). Among these, the most frequently identified genes were *RYR1* in patients with CNM (*n* = 17) [33, 34] or dusty core disease (*n* = 2) [35], and *TTN* (*n* = 6) in patients with CNM [36]. These novel genotype–phenotype associations were reported by us or others during the course of this study. The remaining six families had pathogenic variants in genes usually associated with muscular dystrophies (*HNRNPDL* [37]*, TOR1AIP1 *[38]*, SGCG*, *COL6A2*, *CAV3*, *TRIP4* [39, 40]). These variants were identical or affecting the same residues as described in previously reported dystrophy cases, highlighting the genetic and clinical heterogeneity of CMs.
- Pathogenic variants in known genes associated with other neuromuscular or cardiac diseases: interestingly, three families had novel pathogenic variants in genes usually associated with neuropathies: *ASCC1* (*n* = 2) associated with a severe myopathy with arthrogryposis and bone fractures [41] and *HSPB8* (*n* = 1) linked to a vacuolar adult-onset myopathy [42]. In the latter case, electromyography showed no evidence of an underlying neuronopathy or polyneuropathy. In addition, four families with muscle biopsies showing characteristic intermyofibrillar network and centralized nuclei or core-like structures had pathogenic variants in *CACNA1S*, a gene usually associated with hypokalemic periodic paralysis or malignant hyperthermia [11]. Moreover, four families with CM carried pathogenic variants in *GFPT1, CHRNA1,* or *CHRND*, all three previously associated with congenital myasthenia often involving muscle weakness similar to CM. In contrast to the reported *GFPT1, CHRNA1,* and *CHRND*-related myasthenia families, the muscle biopsies from our patients displayed either protein aggregates and rimmed vacuoles or CNM-typical nuclear centralization. Lastly, pathogenic variants in *MYPN* previously described in patients with cardiomyopathy was found in two patients with nemaline and cap myopathies [43].
- Pathogenic variants in known genes associated with other syndromes: four families presented a pathogenic variant in a gene not previously known to cause specific neuromuscular or cardiac diseases. In one family, we detected a known homozygous pathogenic variant in *IDUA*, associated with a mild form of mucopolysaccharidosis known as Scheie syndrome [44]. In addition to Scheie-typical features as glaucoma, clouded cornea, carpal tunnel syndrome, and aortic valve disease, our patients also presented with distal retractions and cores on the muscle biopsies. In a second family with CNM, a homozygous pathogenic variant was found in the *OPA1* gene, which encodes a mitochondrial protein previously linked to optic atrophy. In the third family, two deceased fetuses diagnosed with CNM had a hemizygous pathogenic variant in the *FOXP3* gene linked to a syndrome with immunodysregulation, polyendocrinopathy, and enteropathy. In the last family, one patient with a pathogenic *EXOSC3* variant presented with pontocerebellar hypoplasia. The muscle biopsy showed not only marked fiber type grouping, suggesting a neurogenic process, but also the presence of cap-like structures and mini-rods [45]. Overall, except for the two *FOXP3* fetuses, the phenotypes of the listed families conformed with the identified pathogenic variants and genes, and our investigations additionally uncovered an abnormal skeletal muscle structure that may have been overlooked in the previously reported families.

**Table 2:** Genes linked to atypical phenotypes. Genes and associated phenotypes, according to the OMIM database, are indicated in the first two columns. Starting from the first row, the genes are grouped into myopathy/ muscle dystrophy genes (*n* = 18), cardiomyopathy genes (*n* = 1), congenital myasthenia genes (*n* = 3), channelopathy genes (*n* = 1), neuropathy genes (*n* = 2), and genes associated with other syndromes (*n* = 4). The new phenotypes found in the MYOCAPTURE cohort are indicated in the third column. EM electron microscopy

Gene	Usual phenotype(s) (OMIM)	New phenotype(s)
Congenital myopathy or muscle dystrophy		
*ACTA1*	Nemaline myopathy	Nemaline myopathy with enlarged perinuclear space (EM)
*CAV3*	Distal myopathy	Tubular aggregates myopathy
*CFL2*	Nemaline myopathy	Centronuclear myopathy
*COL6A2*	Bethlem myopathy/Ulrich congenital muscular dystrophy	Cores myopathy
*FLNC*	Distal myopathy/myofibrillar myopathy	Nemaline myopathy
*HNRNPA2B1*	Inclusion body myopathy with early-onset Paget disease with or without frontotemporal dementia 2	Early onset form of oculopharyngeal muscular dystrophy
*HNRNPDL*	Muscular dystrophy, limb-girdle	Autophagic rimmed vacuolar myopathies
*TPM3*	CAP myopathy/congenital myopathy with fiber type disproportion/nemaline myopathy	Cores myopathy / Centronuclear myopathy / Caps and rods myopathy
*FHL1*	Emery-Dreifuss muscular dystrophy/reducing body myopathy/myopathy, X-linked, with postural atrophy/scapuloperoneal myopathy	Centronuclear myopathy
*MTM1*	Centronuclear myopathy	Congenital fiber-type disproportion
*MYH7*	Laing distal myopathy/myosin storage myopathy/myopathic scapuloperoneal syndrome	Central core myopathy
*RYR1*	Congenital myopathy with susceptibility to malignant hyperthermia/core myopathy/King–Denborough syndrome	Centronuclear myopathy / Dusty core disease
*SGCG*	Muscular dystrophy, limb-girdle, autosomal recessive 5	Myopathy with broad A band disease
*SPEG*	Centronuclear myopathy	Myopathy with no centralized nuclei
*TOR1AIP1*	Muscular dystrophy, autosomal recessive, with rigid spine and distal joint contractures	Centronuclear myopathy
*TRIP4*	Congenital muscular dystrophy, Davignon-Chauveau type/spinal muscular atrophy with congenital bones fractures 1	Centronuclear myopathy with cores
*TTN*	Muscular dystrophy, limb-girdle/myofibrillar myopathy/Salih myopathy/tibial muscular dystrophy tardive	Centronuclear myopathy
Cardiomyopathy		
*MYPN*	Cardiomyopathy	Caps myopathy
Congenital myasthenia		
*CHRNA1*	Congenital myasthenic syndrome type 1A or 1B	Centronuclear myopathy
*CHRND*	Congenital myasthenic syndrome type 3A, 3B or 3C	Centronuclear myopathy
*GFPT1*	Congenital myasthenia with tubular aggregates	Rimmed vacuole myopathy
Channelopathy		
*CACNA1S*	Hypokalemic periodic paralysis type 1	Myopathy with myofibrillar disorganization
Neuropathy		
*ASCC1*	Spinal muscular atrophy with congenital bone fractures	Congenital myopathy
*HSPB8*	CMT type 2L/neuronopathy, distal hereditary, type IIA	Vacuolar myopathy
Other syndromes		
*EXOSC3*	Short stature, hearing loss, retinitis pigmentosa, and distinctive facies (SHRF)	Myopathy with caps and mini-rods
*FOXP3*	Immunodysregulation, polyendocrinopathy, and enteropathy, X-linked	Centronuclear myopathy
*IDUA*	Mucopolysaccharidosis Ih, Ih/s, Is	Central core myopathy
*OPA1*	Behr syndrome/optic atrophy/optic atrophy plus syndrome	Centronuclear myopathy

### New genes implicated in myopathies

As discussed above, we reported the first pathogenic variants causing a primary myopathy in four genes previously linked to other neuromuscular or cardiac diseases: *ASCC1*, *HSPB8*, *CACNA1S,* and *MYPN*. Moreover, in the MYOCAPTURE project, pathogenic variants were found in 10 new genes not previously linked to a neuromuscular genetic disease in 16 families, including *ACTN2*, *CASQ1*, *GGPS1*, *MAP3K20*/*ZAK*, *ORAI1*, *MYO18B*, *PYROXD1*, *SRPK3*, *STIM1,* and *UNC45B* (Tables 2 and 3). The genetic and functional validations evidencing the pathogenic impact of the identified variants in cell and animal models were published previously. An overview is provided in Table 3.

**Table 3:** New genes found implicated in myopathies. New genes and associated phenotypes are indicated in the first three columns. The references for the characterization of the pathogenic variants found in the MYOCAPTURE cohort are indicated in the fourth column

New genes	Clinical description	Histological phenotype	Reference(s)
*STIM1*	Generalized slowly progressive muscle weakness with variable age of onset, Stormorken syndrome	Tubular aggregate myopathy	[20, 46, 47]
*ORAI1*	Generalized slowly progressive muscle weakness with variable age of onset	Tubular aggregate myopathy	[21, 48, 49]
*CASQ1*	Generalized slowly progressive muscle weakness, post-exercise myalgia	Tubular aggregate myopathy	[22, 50]
*PYROXD1*	Generalized slowly progressive muscle weakness	Myopathy with internalized nuclei and myofibrillar disorganization	[23, 51]
*MAP3K20*	Generalized slowly progressive muscle weakness accompanied by decreased vital capacities	Centronuclear myopathy	[24]
*UNC45B*	Generalized slowly progressive muscle weakness accompanied by decreased vital capacities	Myopathy with eccentric cores, internalized nuclei and myofibrillar disorganization	[25]
*MYO18B*	Congenital myopathy with cardiomyopathy and dysmorphic features	Nemaline myopathy	[26, 52]
*ACTN2*	Generalized slowly progressive muscle weakness accompanied by decreased vital capacities	Core myopathy with jagged Z-lines	[27]
*GGPS1*	Muscular dystrophy with hearing loss and ovarian insufficiency	Muscular dystrophy	[28]
*SRPK3/TTN*^a^	Generalized slowly progressive muscle weakness with cardiomyopathy	Core myopathy	[29]

^a^Digenism

In three families with TAM, we found dominant missense variants in *STIM1*, encoding a reticular Ca^2+^ sensor [20, 46, 47, 53]. Together with the plasma membrane Ca^2+^ channel ORAI1, STIM1 forms the key element of the ubiquitous store-operated Ca^2+^ entry (SOCE) pathway, an essential mechanism regulating Ca^2+^ homeostasis in all cell types. The pathogenic *STIM1* variants affected conserved amino acids in the Ca^2+^-binding EF-hands, and functional experiments evidenced a gain-of-function effect leading to SOCE overactivation and excessive extracellular Ca^2+^ entry. As second and third TAM genes, we and others identified pathogenic variants in *ORAI1* [21, 47–49] and in the muscle-specific reticular Ca^2+^ buffer calsequestrin (*CASQ1*) [22, 50].

In two families, pathogenic variants were found in the *PYROXD1* gene, resulting in CM with muscle fibers exhibiting multiple internal nuclei and cores, along with myofibrillar disorganization [23, 51]. PYROXD1 acts as an oxidoreductase, and tests in the yeast model have shown that the identified variants impair the enzymatic activity and enhance the sensitivity of the cells to oxidative stress. For one family, pathogenic *MAP3K20/ZAK* variants were associated with CNM and rimmed sarcolemma, implicating a novel kinase of unknown function in muscle [24]. *UNC45B* encodes a myosin-directed chaperone essential for sarcomeric organization and muscle function. Pathogenic variants in this gene were found in one family with CM, presenting a muscle biopsy showing internalized nuclei and myofibrillar disorganization [25]. In a single family with severe NM and cardiomyopathy, we detected a homozygous truncating variant in *MYO18B*, coding for an unconventional myosin [26, 52]. In one family, a pathogenic *ACTN2* variant induced core myopathy with jagged z-lines. *ACTN2* encodes alpha-actinin 2, a known regulator of the Z-line and sarcomeric structure [27]. In addition, a pathogenic *GGPS1* variant was identified in one family with muscle dystrophy, hearing loss, and ovarian insufficiency syndrome, suggesting an impairment in geranylgeranylation of small GTPases with multisystemic effects [28]. Lastly, digenic inheritance with co-occurrence of heterozygous truncating variants in *TTN* and on the X-chromosomal *SRPK3* gene, encoding a protein kinase, was found in three families with core myopathy [29].

### Widening the genetic and clinical heterogeneity

**Fig. 4:**
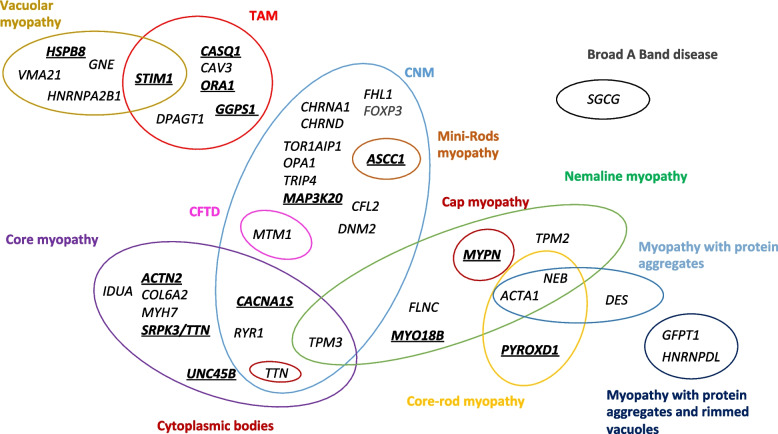
Congenital myopathy-causing genes. In total, pathogenic variants in 48 genes have been identified in the MYOCAPTURE cohort. The diagram depicts the distribution of these genes based on histological phenotypes and the intersections between phenotypes. The new genes not previously linked to a congenital myopathy are indicated in bold and underlined. CFTD congenital fiber type disorder, CNM centronuclear myopathy, TAM tubular aggregate myopathy

Through genetic analysis of this cohort, we were able to widen the known genetic and clinical–histological heterogeneity of CM. Regarding the phenotypic heterogeneity associated with a specific gene, we observed a genetic overlap among the core, nemaline, and CNM cohorts (*CACNA1S*, *RYR1*, *TPM3*, and *TTN*), whereas less common structural anomalies (e.g., TAM) tend to involve other genes (Fig. 4). Such overlap appeared mainly due to the fact that single patient depicted a mix of several structural defects previously associated with distinct cohorts (e.g., cores with mislocalized nuclei linked to pathogenic *RYR1* or *CACNA1S* variants), rather than different patients mutated in the same gene displaying different and specific histopathological hallmarks (e.g., only cores or only central nuclei). Particularly noteworthy is the observation that patients with pathogenic variants in *TTN*, *ACTA1*, *NEB,* or *TPM3* exhibit the widest diversity of histopathological hallmarks in muscle biopsies.

The genetic heterogeneity, i.e., several genes linked to a similar phenotype, is mainly due to (1) the identification of novel myopathy genes and (2) the identification of novel genotype–phenotype associations. For example, at the beginning of the MYOCAPTURE project in 2009, no gene was associated with TAM. This project and other studies identified *STIM1*, *ORAI1,* and *CASQ1* as causative genes [20–22, 48–50, 53]. Another example is the CNM cohort where many genes have been linked, probably in part due to the presence of mislocalized nuclei in the biopsy of patients.

All these findings should be valuable for geneticists, histopathologists, and clinicians for the future diagnosis of different forms of myopathies.

## Discussion

The primary goal of this MYOCAPTURE project was the identification of novel genes associated with CM and the shortening of diagnostic odysseys and dead-ends. It was mainly based on exome sequencing of 310 families with genetically undiagnosed CM, classified in homogeneous cohorts based on clinical and histopathological findings. Pathogenic variants were identified in 50% of the families, of which 79% obtained a definite diagnosis. For the remaining 21%, we identified sequence variants with likely pathogenic effect in muscle genes. Strikingly, only 36% of the patients with resolved diagnosis were linked to a known myopathy gene with a concordant phenotype, pointing out that clinical and histological assessments were not sufficient to anticipate molecular diagnosis in the majority of cases. This included genes previously associated with other myopathies, neuromuscular diseases, or other syndromic disorders. Moreover, the MYOCAPTURE project highlighted the wide genetic and phenotypic heterogeneity characterizing congenital myopathies and successfully identified 14 novel genes not previously associated with muscle diseases.

### Impact of massive parallel sequencing on the diagnosis of congenital myopathies

Patients included in the MYOCAPTURE cohort from 2009 to 2018 were devoid of a genetic diagnosis despite more or less extensive genetic studies. Using exome sequencing, we identified causative variants in 50% of the cases. To confirm the implication of the identified variants and genes, we performed complementary analyses such as segregation studies on DNA samples from relatives or sequencing of muscle RNA. However, a subset of patients was singletons without available parental DNA for segregation studies or biological material for RNA extraction, so that a definite molecular diagnosis could not be delivered. This success rate is similar to other studies using large gene panels or exome sequencing in muscle-related diseases as limb-girdle muscular dystrophies, myopathies, or arthrogryposis multiplex congenital [54–57]. We demonstrate the massive parallel analysis of all coding genes through exome sequencing is more efficient than previous genetic investigations on a gene-by-gene basis or gene panels to pinpoint the genetic defect. Pathogenic variants in *RYR1*, *TTN*, *NEB,* and *ACTA1* were prevalent in our CM cohort, and several reasons account for this. First of all, all four are major CM genes coding for essential proteins of muscle function and structural maintenance, and pathogenic variants are likely to interfere with normal myofiber contractility. Second, the first three belong to the largest genes of the human genome and have a higher probability to accumulate pathogenic DNA variants compared with smaller genes. Third, the large size of the genes precluded efficient variant detection with previously used diagnostic methods like exon-by-exon Sanger sequencing. Fourth, especially *NEB* and *TTN* contain repetitive regions impeding routine sequencing and the assignment of identified variants to specific exons. And lastly, internalized nuclei are commonly seen on muscle biopsies from patients with pathogenic *RYR1* and *TTN* variants, and the major CNM expertise of our laboratory may have driven clinicians and histopathologists to send us a high number of patients with abnormal nuclear positioning on muscle sections.

To our own surprise, only 36% of the families with resolved molecular diagnosis harbored pathogenic variants in known myopathy genes corresponding to their clinical presentation, highlighting a strong genetic and clinical–histological heterogeneity. Conversely, 64% of the pathogenic variants would have been missed with diagnostic panels targeting only a distinct subtype of myopathies. Larger panels encompassing all genes implicated in congenital myopathies or neuromuscular disorders would presumably have found these variants, but they are inapt to discover novel genes. Within the MYOCAPTURE project, we found 14 novel genes (20% of resolved cases) not previously associated with muscle disorders and thereby provided a molecular diagnosis to patients and families that remained without conclusive result after gene panel sequencing. Nonetheless, exome sequencing does not cover most intronic and intergenic regions and only partially and indirectly uncovers structural anomalies as large deletions, inversions, or chromosomal rearrangements impacting on gene expression or mRNA splicing. To overcome these limitations and to ensure a uniform coverage of all coding and non-coding areas, genome sequencing in combination with muscle RNA-seq, and/or other omics approaches can be used [58]. Also, we sequenced DNA extracted mainly from the blood, which precluded in most cases the identification of mosaic mutations present only in a few tissues including muscles. Another limitation of this study is the lack of MRI data that may have allowed a better diagnosis orientation [59, 60]. For example, characteristic involvement patterns of specific muscles and muscle groups have been documented in NM, enabling the differentiation of *NEB*-, *ACTA1*-, and *TPM3*-related NM [61].

Despite all limitations, the MYOCAPTURE project resolved a large number of undiagnosed cases and provided a significant help to the affected families. Indeed, numerous patients obtained the clinical diagnosis of a myopathy several years and decades ago, but still awaited molecular diagnosis. As an example, a patient with recessive *RYR1* variants underwent extensive genetic tests and gene-by-gene investigations over 15 years without conclusive result, and another patient with pathogenic *CACNA1S* variant was clinically followed over 60 years before enrolling our exome sequencing program (family 1 in Schartner et al. [11]). The knowledge of the causative gene enables an adapted genetic counseling for the patients and his or her relatives and also allows a prognosis on disease development in certain cases. It can also suggest complementary examinations and improve clinical follow-up as heart monitoring in *MYH7* or *TTN*-related congenital myopathies or as adaptation of anesthetics for *RYR1* patients. Notably, two unrelated families (family 72 and 76) had a triplet of *RYR1* variants in cis, which was previously linked to malignant hyperthermia susceptibility [62]. In some cases, treatments such as acetylcholinesterase inhibitors may be considered, particularly in patients with pathogenic variants in congenital myasthenia genes (*CHRNA1*, *CHRND, DPAGT1,* and *GFPT1*). Moreover, patients with resolved genetic diagnosis can now be included into clinical trials.

### Refinement of myopathy classification reveals extensive phenotypic and genetic heterogeneity

We found a genetic diagnosis linking a typical phenotype with a gene previously associated to a myopathy in only 36% of resolved cases. The other two-third of cases included genes previously linked to other myopathies (37%; Fig. 3), to congenital myasthenia syndrome (3.5%), to other syndromes (3.5%), or to novel genes (20%). *TTN*, *RYR1,* and *TPM3* are the genes linked to the highest diversity of phenotypes. Overall, we cannot exclude an inclusion bias leading to either the enrichment of patients with a rather variable spectrum of clinical and histological features in some cohorts or to cohorts with less stringent classification criteria. Most biopsies were examined by the Neuromuscular Morphology Unit of the Institute of Myology in Paris to prevent high heterogeneity in histopathological classification. However, other centers have also included patients in the MYOCAPTURE project and may not have followed the same inclusion criteria. For example, a large number of patients enrolling the MYOCAPTURE project exhibited nuclear mislocalization on muscle biopsies and were classified as CNM by the referring clinicians. Although CNM is genetically heterogeneous with six causative genes reported to date (*BIN1*, *DNM2*, *MTM1*, *RYR1*, *SPEG*, *TTN*), exome sequencing identified pathogenic variants in a markedly higher diversity of genes. Some histopathologists may define CNM by the presence of a large number of hypotrophic myofibers with central nuclei, while others may consider a few internal nuclei in normally sized myofibers [33, 34, 63].

In some cases, additional investigations might have been required to fully validate the classification of patients into a specific cohort at the timepoint of inclusion into the MYOCAPTURE project. By way of example, electron microscopy was not performed for all patients (for example to validate the classification of TAM by the detection of tubular membrane aggregates). Indeed, following the genetic diagnosis, some patients could have been re-classified into a different phenotypic cohort.

A major finding was an overlap of histopathological hallmarks in a subset of patients, as the presence of cores with mis-localized nuclei and myofibrillar disorganization for *CACNA1S* patients [11], core-like area, central nuclei and myofibrillar disorganization for *TTN* [36], or nemaline rods, cap structures, and internal nuclei for *TPM3* [64].

Conversely, other patients displayed no specific structural defects on the biopsy. Noteworthy, neonates may not fully recapitulate the structural hallmarks at birth as some develop or change with age, challenging the histopathological classification. Lastly, this study also focused on less explored cohorts, as TAM, leading to the discovery of novel genes. In conclusion, exome sequencing of our large cohort encompassing 310 families revealed a larger than expected phenotypic and genetic heterogeneity.

### Novel myopathy genes suggesting new pathomechanisms and therapeutic targets

Through this exome sequencing project, we identified ten new myopathy genes not previously linked to any neuromuscular diseases and four new myopathy genes that were previously associated with other neuromuscular or cardiac diseases. The validation of the implication of these genes resulted from (1) the sampling of several families sharing similar clinical and histopathological findings through international collaborations, (2) confirmation of the impact of pathogenic variants on the RNA, protein and protein function through in vitro experiments, and (3) the demonstration that the impairment of the in vivo gene function correlated with the patient phenotypes.

These novel genes revealed novel pathomechanism underlying different CM. Genes like *CACNA1S* or *ACTN2*, for which the physiological functions are well known, fit with a pathomechanism implicating impaired excitation–contraction coupling or sarcomere disorganization, respectively [27, 65–67]. We also uncovered novel genes with novel functions in skeletal muscle as the oxidoreductase PYROXD1. How and to what extend the impaired enzymatic activity in our patients contributes to the development of the muscle phenotype is currently not understood, but our functional experiments suggested that the myofibers are more sensitive to oxidative stress [23]. In addition, a novel pathway was implicated in myopathies with the discovery of pathogenic variants in *STIM1*, *ORAI1*, and *CASQ1* that are key players of Ca^2+^ homeostasis through the SOCE mechanism [68, 69]. The strict control of Ca^2+^ entry, storage, and release takes a central role in skeletal muscle physiology and contraction [70].

Noteworthy, the first example of digenism in myopathies was provided with the identification of the co-segregation of deleterious variants in *SRPK3* and *TTN* in patients with progressive childhood-onset skeletal muscle myopathy with cores and centralized nuclei [29]. Such a scenario with pathogenic variants in two independent genes needs to be considered if NGS does not disclose a single implicated gene.

Novel myopathy genes represent novel therapeutic targets that may be more amenable to therapies compared to previously known genes. In case of pathogenic loss-of-function variants, gene replacement strategies could be envisaged even in the absence of a detailed knowledge on the pathomechanism. Inversely, the targeted downregulation of genes, mRNA, or proteins can be applied for pathogenic variants involving a gain-of-function. The implication of novel pathological functions triggers the development of novel therapeutic approaches, while the implication of several proteins in the same pathway sustains the validation of common therapies. For example, repurposing of therapies targeting excitation–contraction coupling and Ca^2+^ homeostasis can be envisaged for *CACNA1S* or the SOCE pathway [70], or anti-oxidant modulators for *PYROXD1*-related myopathy. Also, novel drugs and approaches can be developed through pre-clinical studies, as *ORAI1* RNA silencing for STIM1-related TAM [71].

## Conclusions

Our results confirm the efficiency of exome sequencing to provide genetic diagnoses in patients with undiagnosed congenital myopathies, especially when combined with clinical and histopathological data, and access to additional family members. Most of the resolved cases were linked to a known myopathy gene with atypical signs, highlighting that most genetic diagnoses could not be found with targeted gene sequencing restricted to clinical–histological assessments. In addition, we identified 14 new myopathy genes, revealing novel pathomechanisms and potential therapeutic targets. Finally, we observed an important phenotypic and genotypic heterogeneity. Overall, these results facilitated and accelerated the genetic diagnosis of unresolved families, improved health care for several patients, and opened novel perspectives for either repurposing of existing molecules or the development of novel treatments.

### Supplementary Information

Additional file 1: Table S1. Clinical, histological and pathogenic variants for probands from 123 families with signs of congenital myopathy. Variants were classified according to the ACMG standards and guidelines [18]. Genes are listed in alphabetical order. -: data not available, F: female, M: male, LL: lower limbs, UL: upper limbs, N: normal, CFTD: congenital fiber type disproportion, CNM: centronuclear myopathy, TAM: tubular aggregate myopathy, SD: splice donor. Homozygous pathogenic variants are shown in bold. ^1^The probands of families 8 and 49 are third-degree relatives.

Additional file 2: Figs. S1–S3. Fig S1: Diagnostic yield of exome sequencing as a function of the number of included family members. Diagnosis: patients with pathogenic variant(s). Candidate: patients with suspected pathogenic variant(s) in one candidate gene. Fig S2: Diagnostic yield of exome sequencing as a function of the number of included affected family members. Diagnosis: patients with causal pathogenic variant(s). Candidate: patients with suspected pathogenic variant(s) in one candidate gene. Fig S3: Diagnostic yield of exome sequencing with or without muscle biopsy. Diagnosis: patients with causal pathogenic variant(s). Candidate: patients with suspected pathogenic variant(s) in one candidate gene.

## Data Availability

Due to privacy, ethical, and legal issues in France and the European Union and according to the content of our IRB approved project, exome data supporting the findings of this study are not publicly available. The described pathogenic variants and the associated phenotypes supporting the conclusions of this article are available in the ClinVar repository under the submissions SUB14382184 and SUB14484902 (https://www.ncbi.nlm.nih.gov/clinvar). The datasets combining pathogenic variants and phenotypes of the patients are included in the supporting files in a machine-readable format (.xlsx).

## Abbreviations

CNM: Centronuclear myopathy
CM: Congenital myopathy
NM: Nemaline myopathy
SOCE: Store-operated Ca2 + entry
TAM: Tubular aggregate myopathy
