# Supplementary material for: Exome sequencing in undiagnosed congenital myopathy reveals new genes and refines genes–phenotypes correlations
Source: Genome Med. 2024 Jul 9;16:87. doi: 10.1186/s13073-024-01353-0 (PMC11234750; doi:10.1186/s13073-024-01353-0)
Supplement: Supplementary file 2 — Additional file 2: Figs. S1–S3. Fig S1: Diagnostic yield of exome sequencing as a function of the number of included family members. Diagnosis: patients with pathogenic variant(s). Candidate: patients with suspected pathogenic variant(s) in one candidate gene. Fig S2: Diagnostic yield of exome sequencing as a function of the number of included affected family members. Diagnosis: patients with causal pathogenic variant(s). Candidate: patients with suspected pathogenic variant(s) in one candidate gene. Fig S3: Diagnostic yield of exome sequencing with or without muscle biopsy. Diagnosis: patients with causal pathogenic variant(s). Candidate: patients with suspected pathogenic variant(s) in one candidate gene. [file 13073_2024_1353_MOESM2_ESM.pdf]

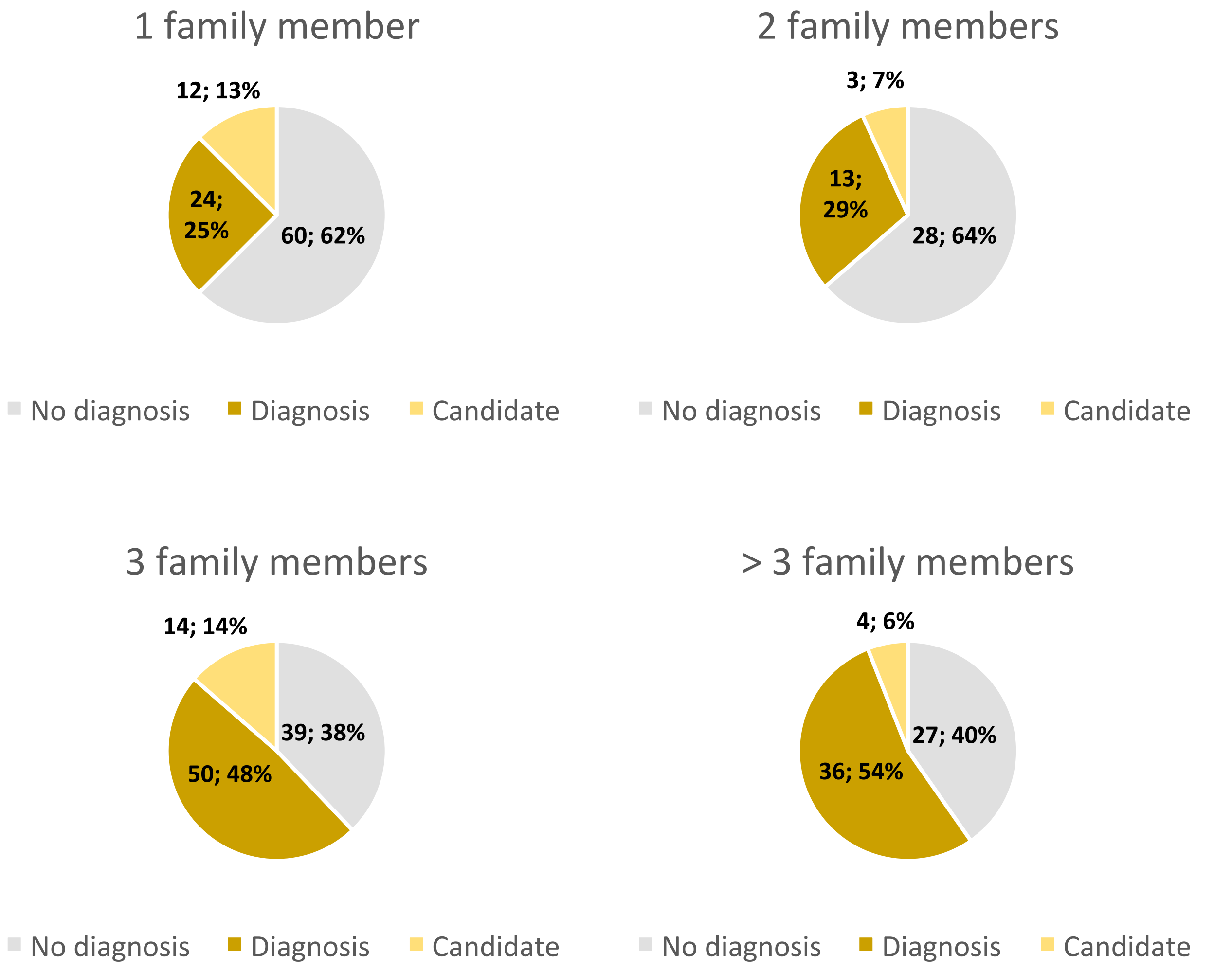

Fig S1: Diagnostic yield of exome sequencing as a function of the number of included family members.

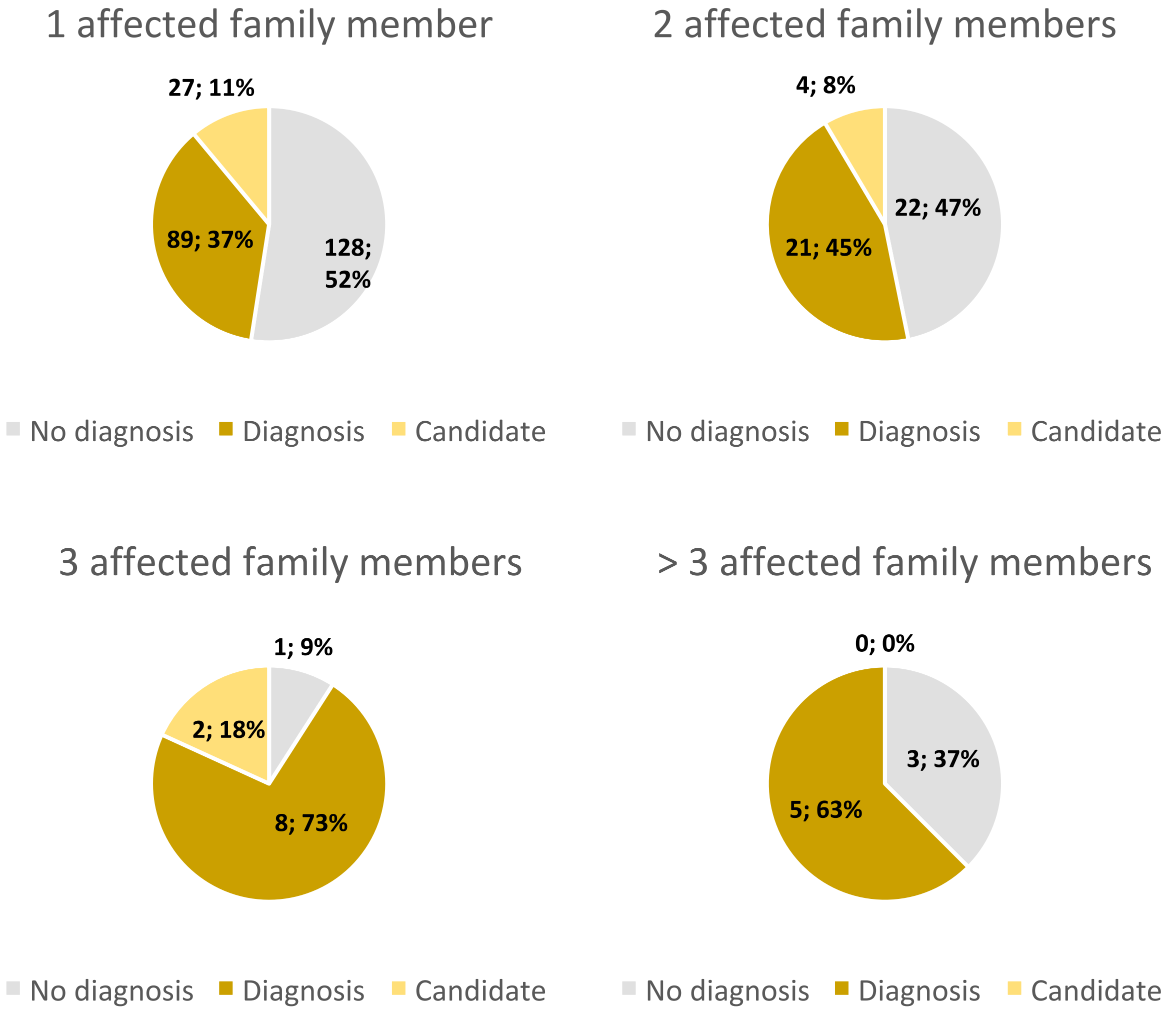

Fig S2: Diagnostic yield of exome sequencing as a function of the number of included affected family members.

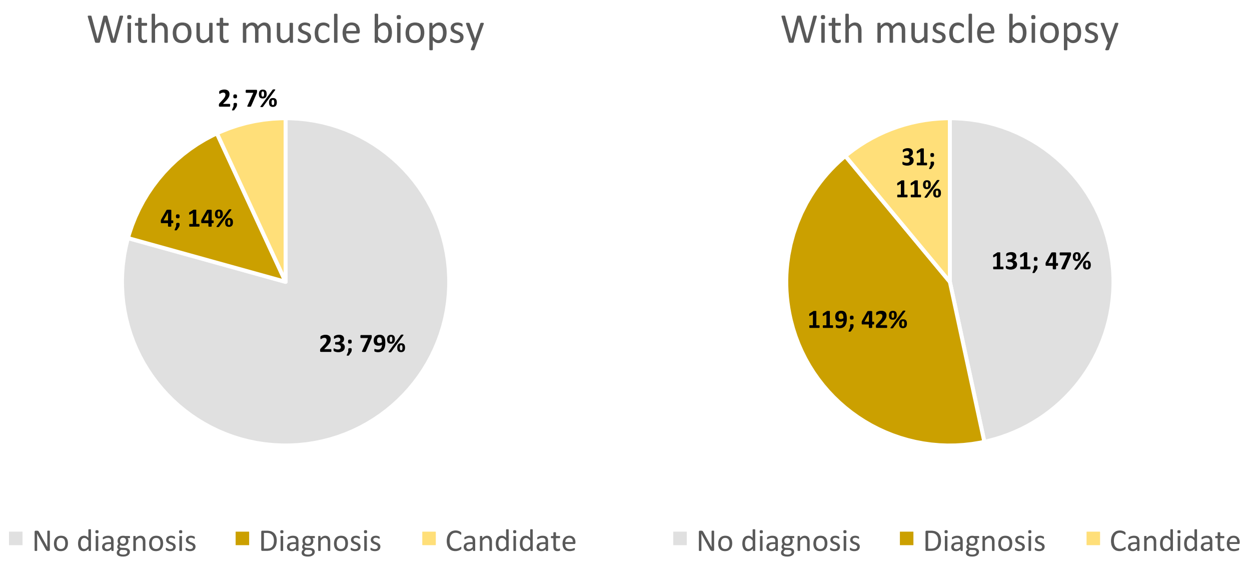

Fig S3: Diagnostic yield of exome sequencing with or without muscle biopsy.
